# Arctigenin Inhibits Osteoclast Differentiation and Function by Suppressing Both Calcineurin-Dependent and Osteoblastic Cell-Dependent NFATc1 Pathways

**DOI:** 10.1371/journal.pone.0085878

**Published:** 2014-01-17

**Authors:** Teruhito Yamashita, Shunsuke Uehara, Nobuyuki Udagawa, Feng Li, Shigetoshi Kadota, Hiroyasu Esumi, Yasuhiro Kobayashi, Naoyuki Takahashi

**Affiliations:** 1 Institute for Oral Science, Matsumoto Dental University, Shiojiri, Nagano, Japan; 2 Department of Biochemistry, Matsumoto Dental University, Shiojiri, Nagano, Japan; 3 Institute of Natural Medicine, University of Toyama, Toyama, Japan; 4 National Cancer Center Hospital East, Kashiwa, Chiba, Japan; Faculdade de Medicina Dentária, Universidade do Porto, Portugal

## Abstract

Arctigenin, a lignan-derived compound, is a constituent of the seeds of *Arctium lappa*. Arctigenin was previously shown to inhibit osteoclastogenesis; however, this inhibitory mechanism has yet to be elucidated. Here, we showed that arctigenin inhibited the action of nuclear factor of activated T-cells, cytoplasmic 1 (NFATc1), a key transcription factor for osteoclastogenesis. NFATc1 in osteoclast precursors was activated through two distinct pathways: the calcineurin-dependent and osteoblastic cell-dependent pathways. Among the several lignan-derived compounds examined, arctigenin most strongly inhibited receptor activator of nuclear factor κB ligand (RANKL)-induced osteoclast-like cell formation in mouse bone marrow macrophage (BMM) cultures, in which the calcineurin-dependent NFATc1 pathway was activated. Arctigenin suppressed neither the activation of nuclear factor κB and mitogen-activated protein kinases nor the up-regulation of c-Fos expression in BMMs treated with RANKL. However, arctigenin suppressed RANKL-induced NFATc1 expression. Interestingly, the treatment of osteoclast-like cells with arctigenin converted NFATc1 into a lower molecular weight species, which was translocated into the nucleus even in the absence of RANKL. Nevertheless, arctigenin as well as cyclosporin A (CsA), a calcineurin inhibitor, suppressed the NFAT-luciferase reporter activity induced by ionomycin and phorbol 12-myristate 13-acetate in BMMs. Chromatin immunoprecipitation analysis confirmed that arctigenin inhibited the recruitment of NFATc1 to the promoter region of the NFATc1 target gene. Arctigenin, but not CsA suppressed osteoclast-like cell formation in co-cultures of osteoblastic cells and bone marrow cells, in which the osteoblastic cell-dependent NFATc1 pathway was activated. The forced expression of constitutively active NFATc1 rescued osteoclastogenesis in BMM cultures treated with CsA, but not that treated with arctigenin. Arctigenin also suppressed the pit-forming activity of osteoclast-like cells cultured on dentin slices. These results suggest that arctigenin induces a dominant negative species of NFATc1, which inhibits osteoclast differentiation and function by suppressing both calcineurin-dependent and osteoblastic cell-dependent NFATc1 pathways.

## Introduction

Arctigenin, a lignan-derived compound, is one of the constituents of the seeds of *Arctium lappa*. Arctigenin is a biologically active lignan with anti-tumor, anti-inflammatory, anti-oxidant, anti-proliferative, and anti-diabetic activities. Arctigenin was shown to block the activation of Akt signaling induced by glucose starvation in pancreatic tumors [1], [2]. Arctigenin also exhibited anti-inflammatory properties against lipopolysaccharide in a macrophage cell line by suppressing inducible nitric oxide synthase expression and mitogen-activated protein (MAP) kinase signals [3], [4]. Arctigenin was recently shown to inhibit osteoclast formation in mouse bone marrow macrophage (BMM) cultures [5]. However, the precise mechanism by which arctigenin inhibits osteoclast development remains to be determined.

Osteoclasts are bone-resorbing multinucleated cells formed from monocyte/macrophage-lineage precursors [6], [7]. Osteoblastic cells such as immature osteoblast-lineage cells, osteoblasts, and osteocytes regulate the differentiation of osteoclast precursors into osteoclasts. Osteoblastic cells express two cytokines that are responsible for osteoclastogenesis: macrophage colony-stimulating factor (M-CSF, also called colony-stimulating factor 1) and receptor activator of nuclear factor κB ligand (RANKL) [8]–[12]. Osteoclast precursors have been shown to express c-Fms (the receptor of M-CSF) and RANK (the receptor of RANKL) and differentiate into osteoclasts in the presence of M-CSF and RANKL [13], [14]. Osteoblastic cells also express osteoprotegerin (OPG), a decoy receptor of RANKL. OPG inhibits osteoclast differentiation by suppressing the interaction between RANKL and RANK. Osteoclasts also express RANK, and the RANKL-RANK interaction was shown to induce the bone-resorbing activity of osteoclasts [6], [7].

A previous study showed that bone-resorption stimulating factors such as 1α,25-dihydroxyvitamin D_3_ [1α,25(OH)_2_D_3_], parathyroid hormone, and prostaglandin E_2_ (PGE_2_) enhanced the expression of RANKL in osteoblastic cells [7]. RANKL activates RANK-mediated signaling, including MAP kinases, nuclear factor κB (NF-κB), c-Fos, and nuclear factor of activated T-cells, cytoplasmic 1 (NFATc1). These RANK-mediated signals induce the expression of osteoclast-related genes such as tartrate-resistant acid phosphatase (TRAP, coded by *Acp5*), cathepsin K (*Ctsk*), osteoclast-associated receptor (*Oscar*), and *Nfatc1* itself [15]–[17].

NFATc1 was identified as a key transcription factor for osteoclastogenesis [15], [18]. The Ca^2+^ oscillation/calcineurin-dependent activation and amplification of NFATc1 in osteoclast precursors are essential for their differentiation into osteoclasts. RANKL induces Ca^2+^ oscillations in osteoclast precursors, and these oscillations activate calcineurin, a Ca^2+^-dependent phosphatase. Activated calcineurin then dephosphorylates multiple serine residues in the NFATc1 protein, which permits the nuclear translocation of NFATc1. NFATc1 in the nucleus acts as a transcription factor for genes specifically expressed in osteoclasts such as *Acp5*, *Ctsk*, *Oscar*, and *Nfatc1* itself. The calcineurin inhibitors, cyclosporin A (CsA) and FK506, have been shown to suppress RANKL-induced osteoclast formation in BMM cultures.

Osteoclastogenesis induced by RANKL also requires co-stimulatory receptor signaling through adaptors containing immunoreceptor tyrosine-based activation motifs (ITAMs). ITAM-containing proteins, such as DNAX-activating protein 12 (DAP12) and Fc receptor common γ chain (FcRγ), facilitate the calcium-mobilizing mechanism during osteoclastogenesis [19]–[22]. Thus, RANK and ITAM signalings cooperated to induce calcium oscillations, resulting in the activation of NFATc1. FcRγ and DAP12 are adaptor molecules that associate with immunoglobulin-like receptors such as OSCAR, triggering receptor expressed on myeloid cells 2 (TREM2), signal-regulatory protein β1 (SIRPβ1) and paired immunoglobulin-like receptor A (PIR-A). OSCAR and PIR-A use FcRγ, while TREM2 and SIRPβ1 associate with DAP12. Recently, Barrow *et al*. reported that OSCAR bound to a specific motif of collagen and stimulated osteoclastogenesis [23]. These results suggest that osteoblastic cells express not only RANKL and M-CSF but also ligands for immunoglobulin-like receptors.

Besides Ca^2+^/calcineurin-dependent signaling, the osteoblastic cell-dependent pathway has also been shown to activate NFATc1 [24], [25]. BMMs obtained from type 2 inositol-1,2,5-triphosphate receptor (IP_3_R2) knockout mice exhibited neither RANKL-induced Ca^2+^ oscillations nor RANKL-induced differentiation into osteoclasts. However, IP_3_R2-deficient osteoclast precursors could normally differentiate into osteoclasts when they were co-cultured with osteoblastic cells. This Ca^2+^ oscillation-independent osteoclastogenesis was shown to be insensitive to FK506 [24]. Kuroda *et al*. recently showed that Cot, a serine/threonine kinase, was involved in the activation and amplification of NFATc1 in the absence of Ca^2+^ oscillations. Cot in osteoclast precursors was activated by cell-cell interactions with osteoblastic cells. Activated Cot increased NFATc1 protein levels through phosphorylation-dependent protein stabilization, thereby amplifying NFATc1 [25]. Cot likely phosphorylates residues of NFATc1 that differ from those targeted by calcineurin-mediated dephosphorylation. Although the osteoblastic cell-derived molecules that activate Cot in osteoclast precursors is unknown, Cot-mediated NFATc1 stabilization contributes to osteoclastogenesis *in vivo*. Mice doubly deficient in DAP12 and FcRγ exhibited severe osteopetrosis owing to the lack of osteoclasts [22]. These results suggest that both calcineurin-dependent and osteoblastic cell-dependent activations of NFATc1 are physiologically important in controlling osteoclast differentiation *in vivo*.

The aim of this study was to elucidate the inhibitory mechanism of arctigenin on the osteoclast differentiation and function. Especially, we examined the intracellular signals including NFATc1 in detail. Arctigenin converted NFATc1 into a lower molecular species, which was translocated in the nucleus of osteoclast-like cells even in the absence of RANKL. Arctigenin inhibited osteoclast-like cell formation not only in BMM cultures, but also in co-cultures of osteoblastic cells and bone marrow cells. In addition, arctigenin suppressed the pit-forming activity of osteoclast-like cells cultured on dentin. These results suggest that arctigenin suppresses both calcineurin-dependent and osteoblastic cell-dependent NFATc1 pathways through the generation of a dominant species of NFATc1.

## Materials and Methods

### Reagents and animals

**Figure 1:**
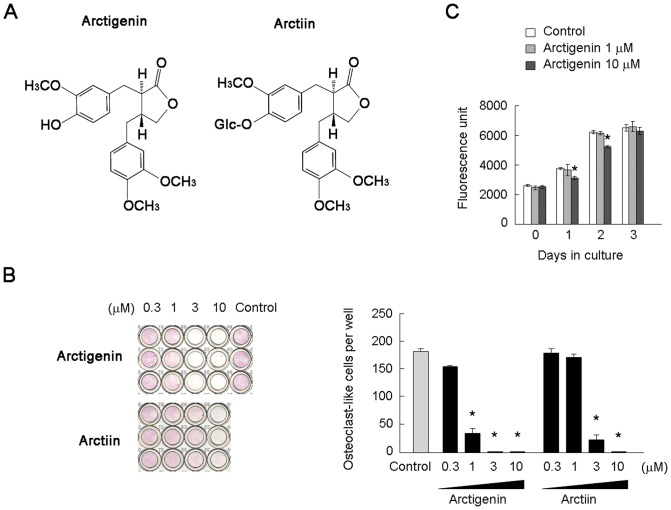
Effects of arctigenin on osteoclast-like cell formation and cell proliferation in BMM cultures. (A) Chemical structures of arctigenin, arctiin, and secoisolariciresinol. (B) Effects of lignan-derived compounds on osteoclast-like cell formation in BMM cultures. BMMs were cultured in 96-well culture plates in the presence of RANKL and M-CSF together with increasing concentrations of the lignan-derived compounds. After cultivation for 3 days, cells were stained for TRAP. TRAP-positive cells appeared as dark red cells. TRAP-positive multinucleated cells containing more than three nuclei were counted as osteoclast-like cells. (C) Effects of arctigenin on the proliferation of BMMs. BMMs were cultured in the presence of M-CSF together with increasing concentrations of arctigenin. Cell proliferation was evaluated on days 0, 1, 2, and 3 using an Alamar Blue assay. The results were expressed as means +/− SD (n = 3). *, p<0.05.

Arctigenin, arctiin, and secoisolariciresinol were purified from *Arctium lappa* and single peaks were confirmed by high performance liquid chromatography. The chemical structures of these compounds were shown in Figure 1A. Human recombinant RANKL was purchased from PeproTech (Rocky Hill, NJ). Human M-CSF (Leukoprol) was obtained from Kyowa Hakko (Tokyo, Japan). 1α,25(OH)_2_D_3_ was from Wako Pure Chemical (Osaka, Japan). CsA, PGE_2_, phorbol 12-myristate 13-acetate (PMA), and ionomycin were from Calbiochem-Sigma (St. Louis, MO). Antibodies for p38, ERK, JNK, IκBα, AKT, and NF-κB p65, and antibodies for phosphorylated forms of p38, ERK, JNK, IκBα, and AKT were from Cell Signaling Technology (Danvers, MA). Antibodies for NFATc1 (7A6), Lamin A/C, and β-actin were obtained from Thermo (Rockford, IL), Santa Cruz (Dallas, TX), and Sigma, respectively. Other chemicals used were of analytical grade. Newborn and 6-week-old ddY mice were purchased from Japan SLC (Shizuoka, Japan).

### Ethics statement

All animal experiments in this study were approved by the Institutional Animal Care and Use Committee of Matsumoto Dental University. All steps were performed to limit suffering in all animal experiments.

### *In vitro* osteoclast-like cell differentiation

Mouse BMMs were prepared as previously described [26]. Briefly, mouse bone marrow cells were cultured for 16 h in α-modified minimal essential medium (α-MEM) with 10% fetal bovine serum (FBS) and 5000 U/mL M-CSF. Non-adherent cells were then collected and used as BMMs. BMMs (3×10^4^ cells/well) for osteoclast formation assay were cultured for 3 days in 96-well culture plates in the presence of 5000 U/mL M-CSF and 100 ng/mL RANKL together with increasing concentrations of lignan-derived compounds or 0.1% dimethyl sulfoxide (DMSO, vehicle control). Osteoblastic cells were obtained form newborn mouse calvariae for the co-culture experiments. Bone marrow cells (1×10^5^ cells/well) and calvarial osteoblastic cells (1×10^4^ cells/well) were co-cultured for 6 days in α-MEM with 10% FBS in the presence of 10 nM 1α,25(OH)_2_D_3_ together with or without test compounds. After cultivation for the indicated periods, cells were fixed and stained for TRAP. TRAP-positive multinucleated cells containing more than three nuclei were counted as osteoclast-like cells. The effects of lignan-derived compounds on the proliferation of BMMs were also evaluated. BMMs (1×10^3^ cells/well) were cultured with increasing concentrations of lignan-derived compounds in 96-well culture plates. The cultures were subjected to the cell viability Alamar Blue assay (Invitrogen, Carlsbad, CA).

### Pit formation assay and actin ring staining

Functionally active osteoclast-like cells were prepared using a mouse co-culture system [27]. Bone marrow cells (1×10^7^ cells) and osteoblastic cells (1×10^6^ cells) were co-cultured in collagen gel-coated 10-cm plates in the presence of 10 nM 1α,25(OH)_2_D_3_ and 1 µM PGE_2_. After a co-culture for 7 days, the collagen-gel plate was treated with collagenase. All cells recovered were suspended in 10 mL of α-MEM containing 10% FBS, and were used as osteoclast-like cell preparations. Osteoclast-like cell preparations were plated on dentin slices (a square piece of 4 mm, 0.75 mm in thickness) in 96-well culture plates (0.2 mL/well) and incubated for 1 h. Dentin slices were then transferred into 48-well culture plates and cultured for an additional 48 h in α-MEM containing 10% FBS (0.5 mL/well) together with or without 1 µM arctigenin. Dentin slices were then recovered, fixed, and stained for TRAP. TRAP-positive cells were counted as osteoclast-like cells. Cells on the slices were removed by cotton swabs and slices were further stained with Mayer's hematoxylin to detect resorbing pits. Resorption pit areas were measured using ImageJ software. To evaluate actin ring formation by osteoclast-like cells, cells on dentin slices were fixed, permeabilized with 0.2% Triton X-100/PBS, and stained with rhodamine-phalloidin (Invitrogen).

### Western blot analysis

Cells were lysed in lysis buffer [20 mM Tris-HCl, pH7.6, 150 mM NaCl, 1 mM EDTA, 50 mM β-glycerophosphate, 1% NP-40, and supplemented with 1× protease inhibitor cocktail (Sigma)], sonicated, and supernatants were collected as total cell lysates. Lysates (20 µg/lane) were electrophoresed on SDS-PAGE gels, transferred to polyvinylidene difluoride membranes, and subjected to immunoblotting using the ECL plus chemiluminescence detection system (GE Healthcare, Buckinghamshire, UK).

### RNA isolation and quantitative RT-PCR

Total RNA was isolated using TRIzol reagent (Invitrogen). Reverse transcription was performed on 1 µg of RNA using oligo(dT) primers and ReverTra Ace reverse transcriptase (Toyobo Life Science, Osaka). cDNA was then amplified using SYBR green Taq RT-PCR reagent (Takara Bio, Shiga, Japan) and expression levels were quantified using the Opticon II real-time PCR instrument (Bio-Rad, Hercules, CA). The primers used for PCR were as follows; *Gapdh*: 5′-TGT GTC CGT CGT GGA TCT GA-3′ (forward) and 5′-TTG CTG TTG AAG TCG CAG GAG-3′ (reverse); *Nfatc1*: 5′-TGG AGA AGC AGA GCA CAG AC-3′ (forward) and 5′-GCG GAA AGG TGG TAT CTC AA-3′ (reverse); *c-Fos*: 5′-CCG AGC TGG TGC ATT ACA GAG A-3′ (forward) and 5′-TGG ATG CTT GCA AGT CCT TGA G-3′ (reverse); *Acp5*: 5′-TTG CGA CCA TTG TTA GCC ACA TA-3′ (forward) and 5′-TCA GAT CCA TAG TGA AAC CGC AAG-3′ (reverse); *Ctsk*: 5′-CAG CAG AAC GGA GGC ATT GA-3′ (forward) and 5′-CTT TGC CGT GGC GTT ATA CAT ACA-3′ (reverse); *Oscar*: 5′-CGT GCT GAC TTC ACA CCA ACA-3′ (forward) and 5′-CAC AGC GCA GGC TTA CGT T-3′ (reverse); *Rankl*: 5′-CAT GTG CCA CTG AGA ACC TTG AA-3′ (forward) and 5′ CAG GTC CCA GCG CAA TGT AAC-3′ (reverse); *Opg*: 5′-CAT GAG GTT CCT GCA CAG CTT C-3′ (forward) and 5′-ACA GCC CAG TGA CCA TTC CTA GTT A-3′ (reverse); *IL-2*: 5′-GCT GTT GAT GGA CCT ACA GGA-3′ (forward) and 5′-TTC AAT TCT GTG GCC TGC TT-3′ (reverse); *GM-CSF*: 5′-GCA TGT AGA GGC CAT CAA AGA-3′ (forward) and 5′ CGG GTC TGC ACA CAT GTT A-3′ (reverse).

### Nuclear extract preparation

Crude osteoclast-like cell preparations were cultured for 4 h on 24-well culture plates. Osteoblastic cells were then removed by washing with 0.1% trypsin/EDTA solution. Purified osteoclast-like cells were further cultured with M-CSF for 16 h. Cells were then incubated for 20 min with 1 µM arctigenin, 1 µg/mL CsA, or 0.1% DMSO (vehicle control) in the presence or absence of 100 ng/mL RANKL. Nuclear and cytoplasmic fractions were prepared using a nuclear extraction kit (Active Motif, Carlsbad, CA) and subjected to Western blot analysis.

### Immunocytochemical localization of NFATc1

The localization of NFATc1 was immunocytochemically examined [28]. Purified osteoclast-like cells on glass coverslips were further cultured for 16 h in the presence of M-CSF, but in the absence of RANKL. Osteoclast-like cells were then incubated for 20 min with 1 µM arctigenin, 100 ng/mL RANKL, or 0.1% DMSO (vehicle control). Cells were fixed with 4% paraformaldehyde, permeabilized with 0.2% Triton X-100/PBS, and blocked with 2% BSA/PBS. An anti-NFATc1 antibody was added to cell preparations, followed by incubation with biotinylated goat anti-mouse IgG and fluorescein-conjugated streptavidin (Vector Laboratories, Burlingame, CA). Nuclei were counterstained with propidium iodide (PI).

### Forced expression of NFATc1

BMMs were retrovirally transduced with NFATc1 cDNA. The constitutively active (ca)-NFATc1 mutant with the alanine substitution of twenty serine residues, which are typically phosphorylated, was shown to be autonomously translocated into the nucleus [29]. The pCX4pur-ca-NFATc1 (constitutively active), pCX4pur-wt-NFATc1 (wild-type), or pCX4pur-GFP control plasmid was transfected into the retrovirus packaging cell line Plat-E [30]. BMMs were incubated for 2 days with virus-rich supernatant in the presence of M-CSF and polybrene. Infected BMMs were further cultured for 3 days with M-CSF and RANKL. Nuclear and cytoplasmic fractions were prepared from infected BMMs as described above, and subjected to Western blot analysis.

### Luciferase reporter assay

The luciferase reporter plasmid pNFAT-Luc, which contained four copies of the NFAT binding site, 5′-GGA GGA AAA ACT GTT TCA TAC AGA AGG CGT-3′, was used (Stratagene-Agilent, La Jolla, CA). BMMs were transfected by electroporation in serum-free solution with Nucleofector II (Amaxa Biosystems-Lonza, Basel, Switzerland). Forty-eight hours after transfection, cells were stimulated with 1 µM ionomycin and 20 nM PMA, in the presence or absence of arctigenin and CsA. Cells were lysed 24 h after stimulation, and luciferase activity was measured with a luciferase assay system (Promega, Madison, WI).

### Chromatin immunoprecipitation (ChIP) assay

BMMs were stimulated with M-CSF and RANKL for 2 days to form mononuclear pre-osteoclast-like cells. These cells were further treated with 100 ng/mL RANKL in the presence or absence of 1 µM arctigenin. Cells were crosslinked with 1% formaldehyde 1 h after the treatment and a nuclear fraction was extracted. The protein-chromatin complex was immunoprecipitated overnight at 4°C with the anti-NFATc1 antibody or control IgG after shearing DNA by sonication. Fragmented DNA were purified and amplified by PCR using primers specific for the *Oscar* promoter [17]: 5′-GAA CAC CAG AGG CTA TGA CTG TTC-3′ and 5′-CCG TGG AGC TGA GGA AAA GGT TG-3′.

### Cultures of osteoblastic cells

Osteoblastic cells were obtained from newborn mouse calvariae as described [27]. Osteoblastic cells were cultured in α-MEM with 10% FBS. Osteoblastic cells were cultured in the presence or absence of 10 nM 1α,25(OH)_2_D_3_ together with or without 1 µM arctigenin for gene expression analysis. The expression of *Rankl* and *Opg* mRNAs was analyzed after cultivation for 24 h by quantitative RT-PCR. Osteoblastic cells were cultured in the presence or absence of 1 µM arctigenin to stain for alkaline phosphatase, a marker of osteoblasts. After cultivation for 6 days, cells were fixed and stained for alkaline phosphatase. Osteoblastic cells were cultured with increasing concentrations of arctigenin for the cell growth assay. The cultures were then subjected to the cell viability Alamar Blue assay (Invitrogen).

### Statistical analysis

All data were expressed as the mean +/− SD. Differences were evaluated by Mann-Whitney's U-test. P values less than 0.05 were considered to be significant. All experiments were independently repeated at least three times and similar results were obtained.

## Results

### Effect of lignan-derived compounds on osteoclast-like cell formation

Arctigenin, arctiin, and secoisolariciresinol are major lignan-derived compounds of *Arctium lappa*. We first compared the inhibitory effects of these compounds on osteoclast-like cell formation in mouse BMM cultures (Fig. 1 and Fig. S1). Mouse BMMs were cultured in the presence of RANKL and M-CSF together with increasing concentrations of these compounds. Osteoclast-like cells formed within 3 days in control cultures. Arctigenin and arctiin inhibited osteoclast-like cell formation in a dose-dependent manner (Fig. 1B). Arctigenin showed stronger inhibitory effects on osteoclast-like cell formation than that of arctiin. Secoisolariciresinol failed to inhibit osteoclast-like cell formation (Fig. S1). Thus, arctigenin showed the strongest inhibitory effect on osteoclast-like cell formation among the three lignans. We next examined the effects of arctigenin on the growth of BMMs (Fig. 1C). Arctigenin had little effect on the growth of BMMs, through 10 µM arctigenin slightly suppressed it on days 1 and 2. The treatment of BMMs with granulocyte-macrophage colony-stimulating factor (GM-CSF) induced their differentiation into dendritic cells. Arctigenin at 1 µM had no inhibitory effect on the dendritic differentiation of BMMs (Fig. S2).These results suggested that the inhibitory effect of arctigenin was specific for osteoclast-like cell differentiation, and was not due to its action on cell growth. Arctigenin at 1 µM inhibited osteoclast-like cell formation more strongly than arctiin did. Therefore, arctigenin at 1 µM was used in further experiments.

### Effect of arctigenin on RANKL-induced signaling in osteoclastogenesis

**Figure 2:**
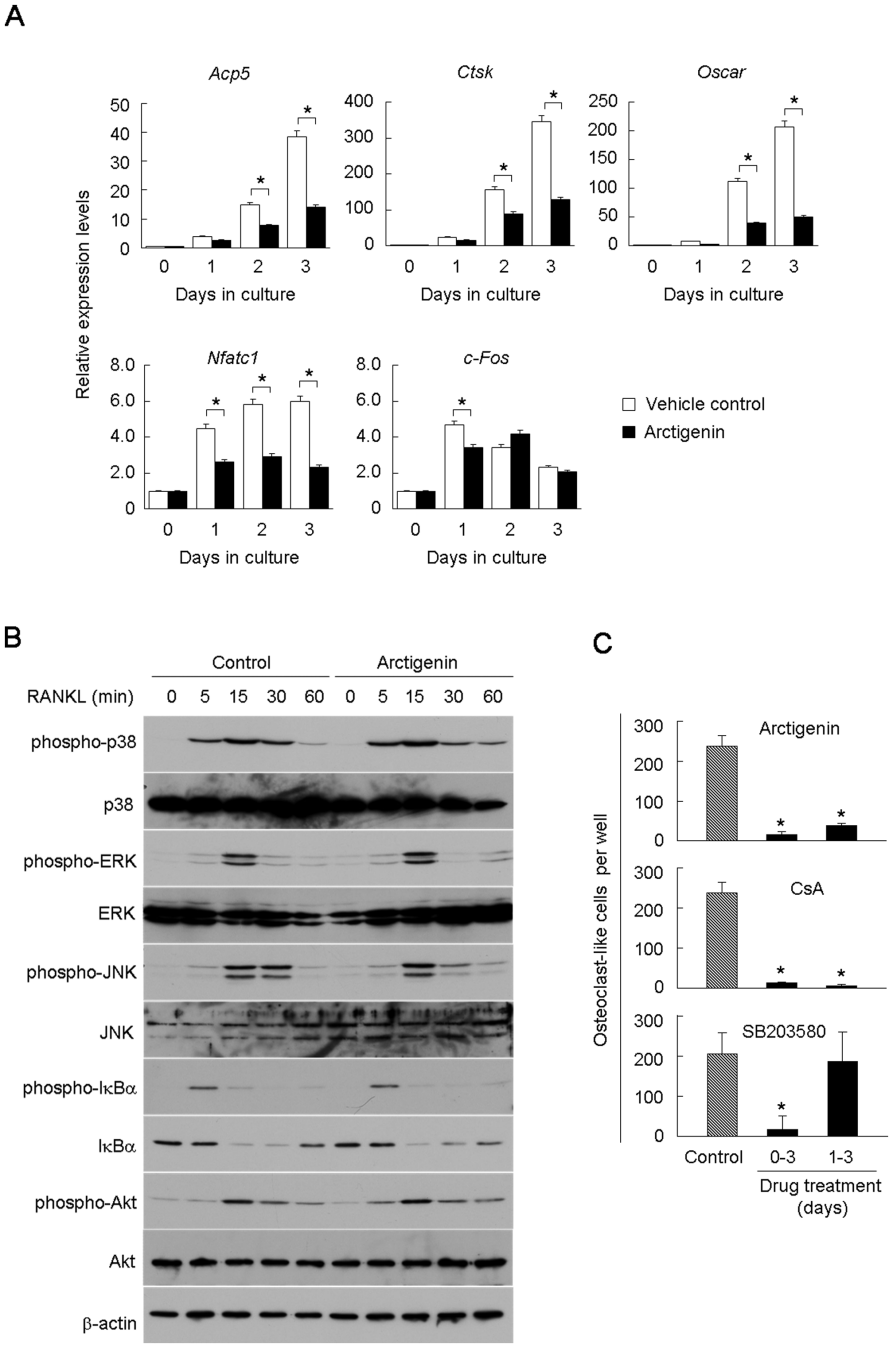
Effects of arctigenin on RANKL-induced signaling in BMMs. (A) Effects of arctigenin on the expression of osteoclast-related genes. BMMs were cultured in the presence of RANKL and M-CSF together with or without 1 µM arctigenin. After cultivation for the indicated periods, the expression of *Acp5*, *Ctsk*, *Oscar*, *Nfatc1*, and *c-Fos* mRNAs was analyzed by quantitative RT-PCR. Expression levels were normalized to *Gapdh* and expressed relative to day 0. Statistical analysis was performed between the control and arctigenin-treated values at indicated time points. (B) Effects of arctigenin on RANKL-induced signals in BMMs. BMMs were pretreated for 10 min with 1 µM arctigenin and further treated with RANKL. Whole cell lysates were harvested and analyzed by Western blotting with the indicated antibodies. (C) Effects of arctigenin, CsA, and SB203580 on osteoclast-like cell formation in BMM cultures. BMMs were cultured for 3 days in the presence of RANKL and M-CSF. BMM cultures were also treated with 1 µM arctigenin, 1 µg/mL CsA, or 1 µM SB203580 during days 0 – 3 and days 1 – 3. Cells were then fixed and stained for TRAP. TRAP-positive multinucleated cells containing more than three nuclei were counted as osteoclast-like cells. The results were expressed as means +/− SD (n = 3). *, p<0.05.

RANKL was previously shown to induce the transcription of osteoclast-related genes such as *Acp5*, *Ctsk*, and *Oscar* by activating NFATc1 [15]. The expression of *Nfatc1* itself was also shown to be up-regulated during osteoclastogenesis [31]. We examined the effect of arctigenin on the expression of these osteoclast-related genes in BMMs during osteoclastogenesis (Fig. 2A). Arctigenin reduced the expression of *Acp5*, *Ctsk*, and *Oscar* at each time point. The expression of the transcription factors, *Nfatc1* and *c-Fos*, was also up-regulated by the treatment with RANKL. The RANKL-induced up-regulation of *Nfatc1* expression, but not that of *c-Fos*, was reduced by arctigenin throughout the experimental period.

We next examined the effect of arctigenin on RANKL-induced signals in BMMs (Fig. 2B). RANKL has been shown to activate NF-κB and MAP kinases, including p38 MAP kinase, ERK, and JNK [32], [33]. Western blot analysis revealed that these signals were similarly activated by RANKL in BMMs in the presence and absence of 1 µM arctigenin (Fig. 2B). A previous study showed that Akt was one of the targets of arctigenin in tumor cells. The phosphorylation of Akt in BMMs was up-regulated by RANKL, and arctigenin had little effect on this phosphorylation (Fig. 2B). These results suggested that arctigenin inhibited other pathways involved in osteoclastogenesis, independent of MAP kinase, NF-κB, and c-Fos signaling.

We next compared the inhibitory action of arctigenin with that of the well-known inhibitors of osteoclastogenesis, CsA (a calcineurin-NFAT inhibitor) and SB203580 (a p38 MAP kinase inhibitor). Arctigenin, CsA, and SB203580 were added to BMM cultures at different time points (Fig. 2C). Osteoclast-like cell formation was inhibited by the addition of these agents during the entire culture period (0-3 days). Arctigenin and CsA, but not SB203580 inhibited osteoclast-like cell formation when they were added 24 h after RANKL stimulation (1–3 days). Thus, signals mediated by p38 MAP kinase appeared to be necessary for the commitment of osteoclast precursors but not for the expression of osteoclast-related markers in the committed cells [34]. The inhibitory action of arctigenin on osteoclastogenesis was similar to that of CsA. These results suggested that NFATc1 was a target molecule of the arctigenin action in BMMs.

### Effects of arctigenin on the transcriptional activity and localization of NFATc1 in osteoclast-like cells

**Figure 3:**
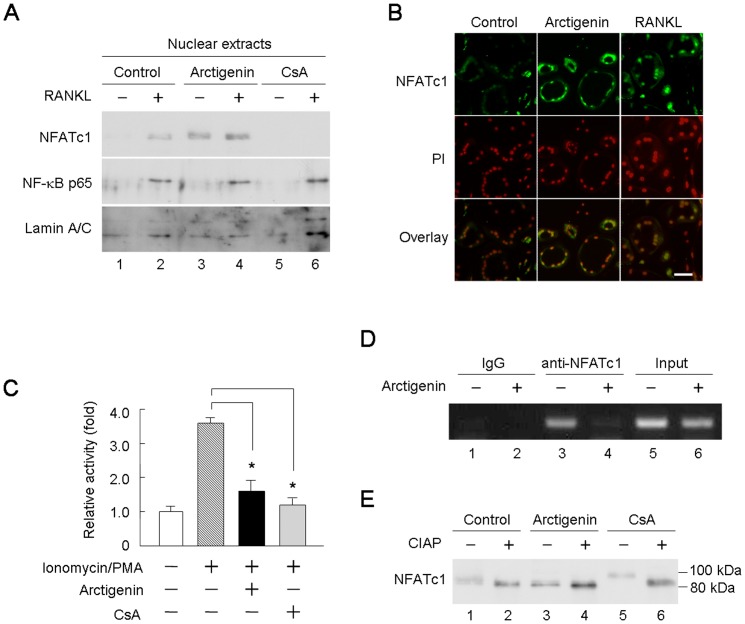
Effects of arctigenin on the nuclear translocation and activation of NFATc1. (A) Effects of arctigenin and CsA on the nuclear translocation of NFATc1 and NF-κB in osteoclast-like cells. Purified osteoclast-like cells were cultured for 16 h in the absence of RANKL. Osteoclast-like cells were then pre-treated for 20 min with or without 1 µM arctigenin or 1 µg/mL CsA, and then further incubated for 20 min with or without RANKL. Nuclear fractions were analyzed by Western blotting with the antibodies indicated. (B) Immunohistochemical detection of NFATc1 in osteoclast-like cells. Purified osteoclast-like cells were cultured on glass plates for 16 h in the absence of RANKL. Cells were then treated for 20 min with or without 1 µM arctigenin or 100 ng/mL RANKL. Cells were fixed and stained for NFATc1 (green). Nuclei were counterstained with propidium iodide (PI, red). Bar = 50 µm. (C) Effects of arctigenin and CsA on NFAT reporter activity. BMMs were transfected with the NFAT-luciferase construct. Forty-eight hours later, cells were incubated for an additional 24 h with or without ionomycin and PMA. Some cultures were treated with 1 µM arctigenin or 1 µg/mL CsA. The fluorescence of the lysates was measured by a luminometer. The results were expressed as means +/− SD (n = 3). *, p<0.05. (D) ChIP assay on the *Oscar* promoter. BMMs were cultured for 2 days in the presence of RANKL and M-CSF. Cells were then treated for 1 h with or without 1 µM arctigenin. Chromatin complexes were immunoprecipitated with the anti-NFATc1 antibody or control IgG. Chromatin DNA fragments were subjected to PCR for the *Oscar* promoter. (E) Western blotting of NFATc1 in osteoclast-like cells. Purified osteoclast-like cells were treated for 10 min with or without 1 µM arctigenin or 1 µg/mL CsA. Cell lysates were then collected. Half of the lysates were treated for 30 min with calf intestine alkaline phosphatase (CIAP). Samples were analyzed by Western blotting with the anti-NFATc1 antibody.

Osteoclast-like cells, which were formed in BMM cultures in the presence of RANKL for 3 days, highly expressed NFATc1 (Fig. 2A). Using purified osteoclast-like cells obtained from co-cultures performed in collagen gel-coated plates, we examined the effect of arctigenin on the nuclear localization of NFATc1 (Fig. 3A). Osteoclast-like cells were cultured overnight in RANKL-free medium. Cells were then treated with RANKL together with or without arctigenin and CsA. Interestingly, NFATc1 was translocated into the nuclei of osteoclast-like cells in response to arctigenin even in the absence of RANKL (Fig. 3A, lane 3). CsA did not show such activity, and inhibited the nuclear translocation of NFATc1 induced by RANKL in osteoclast-like cells (Fig. 3A, lanes 5 and 6). NF-κB p65 was also translocated into the nuclei of osteoclast-like cells in response to RANKL (Fig. 3A, lanes 2, 4, and 6). Neither arctigenin nor CsA inhibited the nuclear translocation of NF-κB p65 (Fig. 3A, lanes 4 and 6). Immunocytochemical analysis confirmed that arctigenin, even in the absence of RANKL, promoted the nuclear localization of NFATc1 in osteoclast-like cells (Fig. 3B). The nuclear localization of NFATc1 was also observed in osteoclast-like cells treated with RANKL.

Treating cells with ionomycin and PMA led to Ca^2+^ oscillations, which induced the nuclear translocation of NFATc1 through the activation of calcineurin. We then examined the effect of arctigenin on the transcriptional activity of NFATc1 in BMMs using a luciferase reporter assay, in which the luciferase gene was driven by the NFAT-binding site (Fig. 3C). The treatment of BMMs with ionomycin and PMA increased NFAT-driven luciferase activity in BMM cultures. Arctigenin as well as CsA suppressed the luciferase activity enhanced by ionomycin and PMA. We then examined whether arctigenin inhibited the recruitment of NFATc1 on the target gene promoters by using a ChIP assay with the *Oscar* promoter (Fig. 3D). BMMs treated with RANKL for 2 days were further cultured in the presence or absence of arctigenin. RANKL stimulation induced the recruitment of NFATc1 on the *Oscar* promoter (Fig. 3D, lane 3), whereas arctigenin inhibited it (Fig. 3D, lane 4). These results indicated that arctigenin inhibited NFATc1 transcriptional activity by suppressing the recruitment of NFATc1 onto the target gene promoter, and also that the mechanism for the inhibitory action of arctigenin on osteoclast formation was different from that of CsA.

NFATc1 is commonly retained in the cytosol as a multi-phosphorylated form. Ca^2+^ oscillations activate calcineurin, which dephosphorylates NFATc1 [29], [30]. We examined the effect of arctigenin on the molecular mass of NFATc1 in osteoclast-like cells (Fig. 3E). NFATc1 in osteoclasts was recognized as a broad band around 90 kDa (Fig. 3E, lane 1). In contrast, NFATc1 in osteoclast-like cells treated with arctigenin was detected as a major band at 85 kDa (Fig. 3E, lane 3). NFATc1 in osteoclast-like cells treated with CsA accumulated as a band at approximately 100 kDa, which may have been due to the hyper-phosphorylation of NFATc1. It was reported that calf intestine alkaline phosphatase (CIAP) dephosphorylated NFATc1 at more than 20 phosphorylated amino acid residues and produced lower molecular weight species of NFATc1 *in vitro* [37]. NFATc1 in these cell lysates shifted to approximately 85 kDa after being treated with CIAP (Fig. 3E, lanes 2 and 4). These results suggest that arctigenin converted NFATc1 into a lower molecular weight species.

### Effect of arctigenin on osteoclastogenesis induced by the forced expression of NFATc1

**Figure 4:**
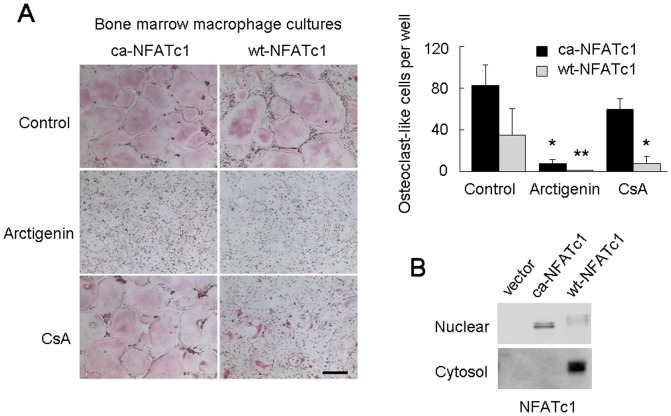
Effect of arctigenin on osteoclast-like cell formation induced by the forced expression of NFATc1. (A) Osteoclast-like cell formation in BMM cultures. BMMs were retrovirally transduced with constitutively active (ca)-NFATc1, wild-type (wt)-NFATc1, or control GFP genes, and cultured for 2 days in the presence of M-CSF. Cells were further cultured in the presence of RANKL and M-CSF together with or without 1 µM arctigenin or 1 µg/mL CsA. After cultivation for 3 days, cells were fixed and stained for TRAP. TRAP-positive multinucleated cells containing more than three nuclei were counted as osteoclast-like cells. The results were expressed as means +/− SD (n = 4). *, p<0.05. **, p<0.01. Bar = 50 µm. (B) Subcellular distribution of NFATc1. Nuclear and cytosol fractions were prepared from BMMs transfected with or without ca-NFATc1 and wt-NFATc1 and analyzed by Western blotting with the anti-NFATc1 antibody.

The constitutively active NFATc1 mutant (ca-NFATc1) was shown to be autonomously translocated into the nucleus [37]. We examined whether the forced expression of ca-NFATc1 could rescue arctigenin-inhibited osteoclastogenesis (Fig. 4). BMMs were retrovirally transduced with ca-NFATc1, wild-type (wt)-NFATc1, and control GFP cDNA. The forced expression of ca-NFATc1, but not wt-NFATc1 in BMMs rescued osteoclast-like cell formation inhibited by CsA (Fig. 4A, lowest panels). In contrast, ca-NFATc1 failed to rescue osteoclastogenesis inhibited by arctigenin. Western blot analysis confirmed that ca-NFATc1 was mainly located in the nucleus of BMMs, whereas wt-NFATc1 was dominantly retained in the cytosol (Fig. 4B). These results suggest that arctigenin did not inhibit the nuclear translocation of ca-NFATc1, but reduced its transcriptional activity. It was also reported that the survival of thymocytes was supported by interleukin 7 (IL-7) through the phosphorylation of Tyr-371 in NFATc1, and the forced expression of Y371F-NFATc1 mutant enhanced the thymocyte survival [38]. Therefore, we examined the effect of the Y371F mutant of NFATc1 on osteoclastogenesis in the presence or absence of arctigenin. The overexpression of Y371F-NFATc1 in BMMs neither induced osteoclastic differentiation nor rescued osteoclastogenesis inhibited by arctigenin (Fig. S3), suggesting that the phosphorylation status of Tyr-371 in NFATc1 is not involved in the inhibitory effect of arctigenin on osteoclastogenesis.

### Effect of arctigenin on osteoclastogenesis induced by the osteoblastic cell-dependent NFATc1 pathway

**Figure 5:**
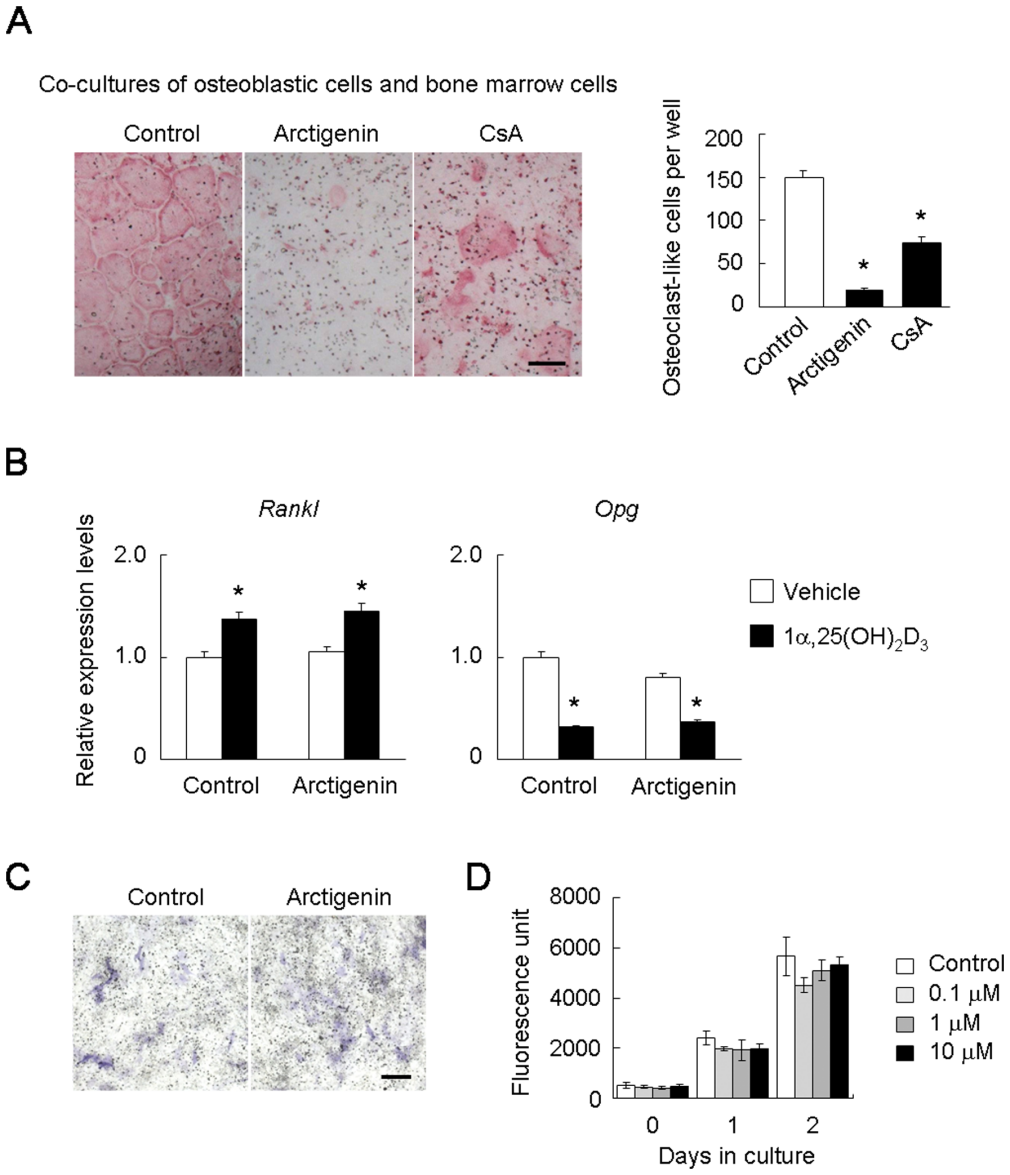
Effect of arctigenin on osteoclast-like cell formation in the co-culture with osteoblastic cells. (A) Effects of arctigenin and CsA on osteoclast-like cell formation in the co-culture. Osteoblastic cells and bone marrow cells were co-cultured in the presence of 1α,25(OH)_2_D_3_ together with or without 1 µM arctigenin or 1 µg/mL CsA. After cultivation for 6 days, cells were fixed and stained for TRAP. TRAP-positive multinucleated cells containing more than three nuclei were counted as osteoclast-like cells. Bar = 50 µm. (B) Effects of arctigenin on RANKL and OPG expression in osteoblastic cells. Osteoblastic cells were cultured for 24 h in the presence or absence of 10 nM 1α,25(OH)_2_D_3_ together with or without 1 µM arctigenin. The expression of *Rankl* and *Opg* mRNAs was analyzed by quantitative RT-PCR. The expression level was normalized to *Gapdh* and expressed relative to the vehicle control. (C) Alkaline phosphatase staining of osteoblastic cell cultures. Osteoblastic cells were cultured for 6 days in the presence or absence of 1 µM arctigenin. Cells were then fixed and stained for alkaline phosphatase. Alkaline phosphatase-positive cells appeared as blue cells. Bar = 50 µm. (D) Effects of arctigenin on the proliferation of osteoblastic cells. Osteoblastic cells were cultured with increasing concentrations of arctigenin. Cell proliferation was evaluated on days 0, 1, and 2 using an Alamar Blue assay. The results were expressed as means +/− SD (n = 3). p<0.05.

NFATc1 has also been shown to be activated by the osteoblastic cell-dependent pathway [24], [25]. We compared the effect of arctigenin on 1α,25(OH)_2_D_3_-induced osteoclast-like cell formation in co-cultures of osteoblastic cells and bone marrow cells with that of CsA (Fig. 5A). Arctigenin strongly inhibited osteoclast-like cell formation in the co-culture, whereas CsA did not abrogate osteoclastogenesis supported by osteoblastic cells. 1α,25(OH)_2_D_3_ increased *Rankl* expression and decreased *Opg* expression in osteoblastic cells, both of which were not affected by arctigenin (Fig. 5B). The appearance of alkaline phosphatase-positive osteoblastic cells in osteoblastic cell cultures was not affected by arctigenin (Fig. 5C). Arctigenin had no effect on the proliferation of osteoblastic cells at the concentrations examined (Fig. 5D). These results suggest that arctigenin directly acted on precursors of osteoclast-like cells in the co-cultures, and inhibited their differentiation into osteoclast-like cells by suppressing the osteoblastic cell-dependent NFATc1 pathway.

### Effect of arctigenin on the pit-forming activity of osteoclast-like cells

**Figure 6:**
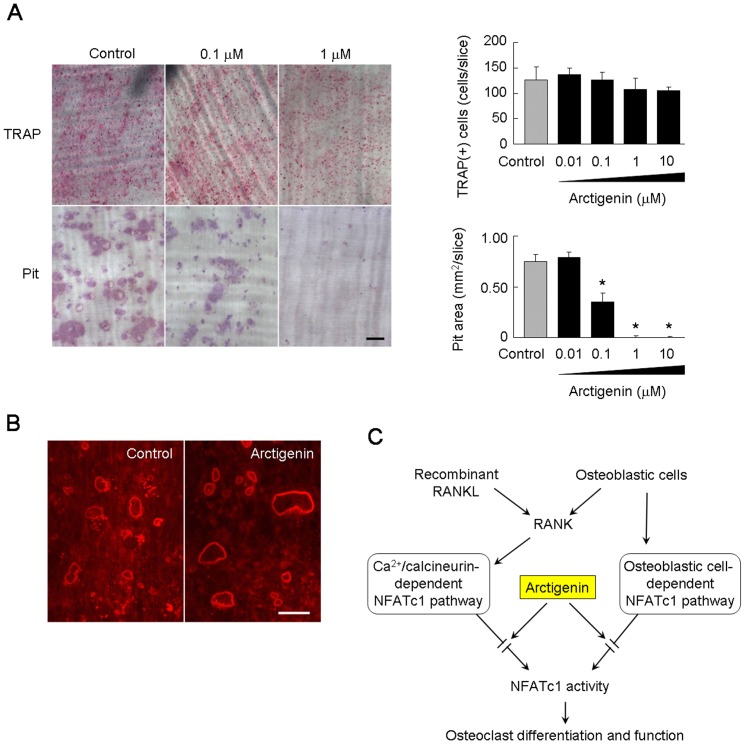
Effect of arctigenin on osteoclast-like cell function. (A) Effects of arctigenin on the pit-forming activity of osteoclast-like cells cultured on dentin slices. Osteoclast-like cell preparations were cultured on dentin slices in the presence or absence of 1 µM arctigenin. After cultivation for 48 h, cells on the slices were fixed and stained for TRAP. TRAP-positive cells were counted as osteoclast-like cells. Cells were then removed from dentin slices, and slices were stained with Mayer's hematoxylin to visualize resorption pits. Resorption pit areas were measured using ImageJ software. The results were expressed as means +/− SD (n = 6). *, p<0.01. Bar = 50 µm. (B) Effect of arctigenin on actin ring formation by osteoclast-like cells. Osteoclast-like cell preparations were cultured on dentin slices in the presence or absence of 1 µM arctigenin. After cultivation for 24 h, cells on the slices were fixed and stained with Rhodamine-phalloidin. Bar = 50 µm. (C) A hypothetical model for the action of arctigenin on the differentiation and function of osteoclasts. Arctigenin inhibited RANKL-induced osteoclast-like cell formation in BMM cultures, in which the Ca^2+^/calcineurin-dependent NFATc1 pathway was activated. Arctigenin also inhibited osteoclast-like cell formation in co-cultures with osteoblastic cells, in which the osteoblastic cell-dependent NFATc1 pathway was activated. The pit-forming activity of osteoclast-like cells was also inhibited by arctigenin. Arctigenin induced a lower molecular weight species of NFATc1, which may act as the dominant negative inhibitor of NFATc1.

There is little conclusive evidence to show that NFATc1 plays an essential role in osteoclast function because an NFATc1 deficiency induces osteopetrosis without osteoclasts. We examined the effect of arctigenin on the pit-forming activity of osteoclast-like cells (Fig. 6). Osteoclast-like cell preparations obtained from co-cultures in collagen gel-coated plates were placed on dentin slices and further cultured for 48 h in the presence of increasing concentrations of arctigenin. The number of TRAP-positive cells on dentin slices after cultivation for 48 h was not significantly changed by the treatment with arctigenin (Fig. 6A), which suggested that the survival of osteoclast-like cells supported by osteoblastic cells was not affected by arctigenin. Cells were then removed, and dentin slices were stained with Mayer's hematoxylin (Fig. 6B). Many resorption pits (purple areas) were formed on the slices in control cultures. The pit-forming activity of osteoclast-like cells was inhibited by arctigenin in a dose-dependent manner. Osteoclast-like cells on bone form actin rings (corresponding to sealing zones) to resorb bone. We examined the effect of arctigenin on actin ring formation by osteoclast-like cells. Osteoclast-like cells cultured on dentin slices formed actin rings even in the presence of arctigenin (Fig. 6C). Thus, arctigenin suppressed the bone-resorbing activity of osteoclast-like cells without affecting their survival or cytoskeletal structures.

## Discussion

We showed that arctigenin inhibited osteoclast-like cell differentiation and function in a dose-dependent manner. Both the differentiation and function of osteoclast-like cells were almost completely inhibited by 1 µM arctigenin. Arctiin also inhibited osteoclast-like cell differentiation, but a higher concentration was needed to achieve the same effect as that of arctigenin. The chemical structure of arctigenin is similar to that of arctiin, a glucoside of arctigenin, but not to secoisolariciresinol. These results suggest that the target molecule of arctigenin and arctiin is the same in BMMs. Arctigenin has been shown to affect various biological reactions *in vitro*. The minimum effective concentration of arctigenin in most reactions was reported to be much higher than 1 µM [4], [39]–[42]. The differentiation of BMMs into dendritic cells was not affected by arctigenin. These results suggest that the target molecule of arctigenin is very specific for osteoclast lineage cells.

Kim *et al*. previously reported that arctigenin suppressed activation of NF-κB, phosphorylation of ERK, and amplification of NFATc1 in BMMs [5]. However, significant suppression of NF-κB and ERK signals by arctigenin was not observed in our cultures. Such a difference may be due to the different experimental conditions employed. The arctigenin-induced suppression of NFATc1 amplification was similarly observed in both Kim *et al*.'s and our experiments. Several lines of evidence in our experiments have indicated that the target molecule of arctigenin was NFATc1 in BMMs and osteoclast-like cells. First, arctigenin specifically suppressed the RANKL-induced up-regulation of NFATc1 in BMMs. Arctigenin had no inhibitory effect on the RANKL-induced activation of NF-κB and MAP kinases or the expression of *c-Fos*. Second, arctigenin as well as CsA could suppress the osteoclastic differentiation of BMMs even when added at the late stage of the culture. BMMs in the late stage of cultures are committed osteoclast precursors, which already express high levels of NFATc1. Third, similar to CsA, arctigenin suppressed the transcription activity of NFATc1 in a luciferase reporter assay. However, the reappearance of IκBα in BMMs treated with RANKL was not affected by arctigenin (Fig. 2B). This suggests that arctigenin does not inhibit the transcriptional activity of NF-κB. Fourth, arctigenin inhibited the RANKL-induced transcriptional activity of NFATc1 on the *Oscar* promoter. Fifth, arctigenin inhibited osteoclast-like cell formation in co-cultures with osteoblastic cells, in which osteoblastic cell-dependent NFATc1 signaling had been activated. In addition, arctigenin completely suppressed the pit-forming activity of mature osteoclast-like cells. Interestingly, these effects of arctigenin were observed at a concentration as low as 1 µM. Taken together, these results suggest that the target molecule of arctigenin is the same in BMMs and mature osteoclast-like cells, and NFATc1 is the most possible target of arctigenin in osteoclast-lineage cells (Fig. 6D).

How does arctigenin inhibit the action of NFATc1 in BMMs and mature osteoclasts? Arctigenin induced the nuclear translocation of NFATc1, but not that of NF-κB in osteoclast-like cells in the absence of RANKL. The molecular weight of NFATc1 in osteoclast-like cells was reduced to approximately 85 kDa by the treatment with arctigenin, but not with CsA, which indicated that arctigenin-induced modification of NFATc1. These results suggested that arctigenin-treated NFATc1 acted as the dominant-negative form of NFATc1. In support of this hypothesis, the forced expression of a ca-NFATc1 mutant failed to rescue arctigenin-inhibited osteoclastogenesis. The amount of force-expressed ca-NFATc1 was much higher than that of endogenous NFATc1 (Fig. 4B). This finding may also indicate that arctigenin inactivate even the ca-NFATc1 mutant. In addition, arctigenin, but not CsA strongly inhibited osteoclast-like cell formation in co-cultures with osteoblastic cells. These results suggest that a dominant-negative form of NFATc1 processed by arctigenin can suppress both calcineurin-dependent and osteoblastic cell-dependent NFATc1 signals.

Arctigenin induced the nuclear localization of 85 kDa NFATc1 in osteoclast-like cells even in the absence of RANKL. In order to determine the mechanism of the production and nuclear localization of 85 kDa NFATc1, we performed two additional experiments. In one experiment, hemagglutinin (HA)-tagged ca-NFATc1 (HA-ca-NFATc1) was expressed in BMMs, which were treated with or without arctigenin. HA-ca-NFATc1 protein was similarly detected as an 85 KDa protein in BMMs even in the presence of arctigenin (Fig. S4). These results suggest that arctigenin treatment does not cause the fragmentation of NFATc1. NFATc1 also shifted to approximately 85 kDa after treatment with CIAP (Fig. 3E). Therefore, it is possible that arctigenin enhances dephosphorylation of NFATc1. We then examined effects of phosphatase inhibitors such as 3,4-dephostatin [protein tyrosine phosphatase (PTP) inhibitor], NSC87877 [SH2 domain-containing inositol phosphatase (SHIP) 1/2 and PTP1B inhibitor], okadaic acid (protein phosphatase 2A inhibitor), and sodium stibogluconate (SHIP1 inhibitor) on the arctigenin-induced conversion of NFATc1. However, those phosphatase inhibitors failed to suppress the arctigenin-induced production of 85 kDa NFATc1 (Fig. S5). Thus, the mechanism of the arctigenin-induced production and nuclear localization of 85 kDa NFATc1 and the structure-function relationship of NFATc1 are still unknown. The mass spectrometry analysis on the NFATc1 protein to determine the phosphorylation status will elucidate the mechanism of arctigenin-induced modifications of NFATc1.

Interleukin 2 (IL-2) and granulocyte-macrophage colony-stimulating factor (GM-CSF) were reported to be the target genes of NFATc1 in T cells [35], [36], [43]. Our preliminary experiments showed that neither the expression of IL-2 nor that of GM-CSF in splenic T cells was inhibited by arctigenin, while CsA suppressed the expression of both genes (Fig. S6). Unlike osteoclasts, T cells have been shown to express NFATc2 and NFATc3 as well as NFATc1 [35], [44]. Therefore, other NFAT family members may compensate for the loss of NFATc1 activity in T cells treated with arctigenin. The differentiation of BMMs into osteoclasts is shown to be tightly regulated by NFATc1. These results suggest that the inhibitory action of arctigenin on NFATc1 is specific for osteoclast lineage cells. The calcinurin-dependent activation of NFATc1 was shown to be necessary for the survival of thymocytes treated with IL-7 [38]. Treatment of thymocytes with IL-7 induced the phosphorylation of Tyr-371 in NFATc1, and the expression of Y371F-NFATc1 enhanced the survival of thymocytes. However, the expression of Y371F-NFATc1 in BMMs failed to rescue arctigenin-induced inhibition of osteoclastogenesis (Fig. S3). These results suggest that the action of arctigenin on NFATc1 signals is regulated differently in osteoclast lineage cells and thymocytes.

Although NFATc1 signaling was previously proposed to be involved in the bone-resorbing activity of osteoclasts, conclusive evidence has not yet confirmed this. The genetic deletion of NFATc1 in the osteoclast lineage cells resulted in a deficiency in osteoclasts [15]. Therefore, difficulties are associated with investigating the precise role of NFATc1 in the bone-resorbing activity of osteoclasts. Komarova et al. demonstrated that local acidification enhanced the bone-resorbing activity of osteoclasts through the Ca^2+^/calcineurin-dependent NFATc1 pathway [45]. Treating osteoclast-like cells with RANKL induced the nuclear translocation of NFATc1 and their pit-forming activity. These results suggest that NFATc1 is also involved in the activation of osteoclast-like cells. We showed that arctigenin inhibited the pit-forming activity of osteoclast-like cells cultured on dentin slices. This inhibitory effect of arctigenin was observed at a concentration as low as 1 µM. Interestingly, arctigenin showed no inhibitory effects on actin ring formation or the survival of osteoclast-like cells. Calcitonin and bisphosphonates, well-known inhibitors of osteoclast function, were shown to disrupt actin rings in osteoclast-like cells [7]. These results suggest that NFATc1 signals are involved in the bone-resorbing activity of osteoclasts without affecting the survival or cytoskeletal structure of osteoclasts (Fig. 6D).

In conclusion, arctigenin inhibited osteoclast-like cell formation in BMM cultures and co-cultures with osteoblastic cells by suppressing NFATc1 signals. Arctigenin appeared to induce the dominant negative species of NFATc1. Thus, arctigenin promises to be a useful agent for investigating the role of NFATc1 in osteoclastic bone resorption. Further experiments will elucidate the molecular mechanism for the inhibitory action of arctigenin on the differentiation and function of osteoclasts.

## Supporting Information

Figure S1 **Effect of secoisolariciresinol on osteoclast-like cell formation.** The chemical structure of secoisolariciresinol was shown. BMMs were cultured in 96-well culture plates in the presence of RANKL and M-CSF together with increasing concentrations of secoisolariciresinol. After cultivation for 3 days, cells were stained for TRAP. TRAP-positive cells appeared as dark red cells. TRAP-positive multinucleated cells containing more than three nuclei were counted as osteoclast-like cells.
(TIF)

Figure S2 **Effect of arctigenin on the differentiation of dendritic cells.** BMMs (1.5×10^5^ cells) were cultured for 1 week in 60-mm dishes in the presence of 20 ng/mL GM-CSF with or without 1 µM arctigenin. Cells were analyzed for the expression of CD11c and CD86 by fluorescence-activated cell scanning. The numbers in the top right corners indicate the percentages of CD11c/CD86 double positive cells as differentiated dendritic cells. Experiments were performed four times, and representative data are shown.
(TIF)

Figure S3 **Effect of arctigenin on osteoclast-like cell formation induced by the forced expression of an Y371F-NFATc1 mutant.** BMMs (3×10^4^ cells) were retrovirally transduced with Y371F-NFATc1 or control GFP, and cultured for 2 days in the presence of 5000 U/mL M-CSF in 96-well culture plates. Cells were further cultured in the presence of 100 ng/mL RANKL and/or 5000 U/mL M-CSF together with or without 1 µM arctigenin. After cultivation for 3 days, cells were fixed and stained for TRAP. TRAP-positive multinucleated cells containing more than three nuclei were counted as osteoclast-like cells. The results were expressed as means +/− SD (n = 4).
(TIF)

Figure S4 **Effect of arctigenin on processing NFATc1.** BMMs (3×10^4^ cells) were retrovirally transduced with a hemagglutinin (HA)-tagged ca-NFATc1 cDNA, and cultured for 2 days in the presence of 5000 U/mL M-CSF in 96-well culture plates. Cells were further cultured in the presence of 100 ng/mL RANKL and 5000 U/mL M-CSF together with or without 1 µM arctigenin. After cultivation for 2 days, total cell lysates were analyzed by Western blotting analysis using an anti-HA antibody or an anti-β-actin antibody.
(TIF)

Figure S5 **Effect of phosphatase inhibitors on the conversion of lower molecular species of NFATc1 induced by arctigenin.** Purified osteoclast-like cells (2000 cells) were cultured in 24-well culture plates in the presence or absence of 10 µM 3,4-dephostatin [protein tyrosine phosphatase (PTP) inhibitor], 10 µM NSC87877 [SH2 domain-containing inositol phosphatase (SHIP)1/2 and PTP1B inhibitor], 0.5 µM okadaic acid (protein phosphatase 2A inhibitor), or 100 µM sodium stibogluconate (SHIP1 inhibitor) together with or without 1 µM arctigenin. After cultivation for 10 min, whole cell lysates were harvested and analyzed by Western blotting using an anti-NFATc1 antibody.
(TIF)

Figure S6 **Effect of arctigenin on the expression of T cell-related genes.** Mouse splenocytes (2×10^6^ cells) were activated for 4 h in the presence of 1 µM ionomycin and 20 nM PMA in 24-well culture plates. Cells were further cultured with or without 1 µM arctigenin and 1 µg/mL CsA. After cultivation for 2 h, *IL-2* and *GM-CSF* mRNA levels were analyzed by quantitative RT-PCR. Expression levels were normalized to *Gapdh* and the values were relative to unstimulated controls. The results were expressed as means +/− SD (n = 3). *, p<0.05; NS, not significant.
(TIF)
