# Supplementary figures and images for: Arctigenin Inhibits Osteoclast Differentiation and Function by Suppressing Both Calcineurin-Dependent and Osteoblastic Cell-Dependent NFATc1 Pathways
Source: PLoS One. 2014 Jan 17;9(1):e85878. doi: 10.1371/journal.pone.0085878 (PMC3895012; doi:10.1371/journal.pone.0085878)

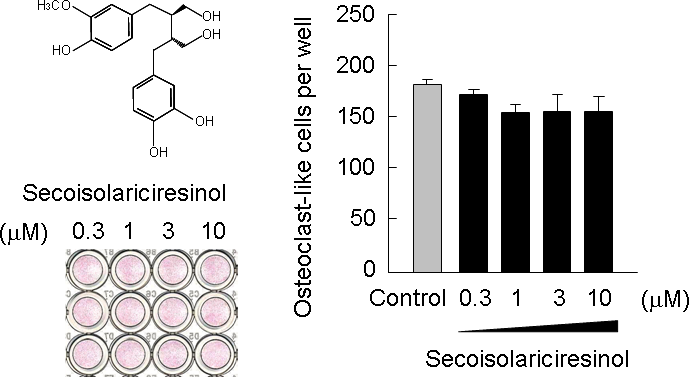

Supplement: Figure S1 — Effect of secoisolariciresinol on osteoclast-like cell formation. The chemical structure of secoisolariciresinol was shown. BMMs were cultured in 96-well culture plates in the presence of RANKL and M-CSF together with increasing concentrations of secoisolariciresinol. After cultivation for 3 days, cells were stained for TRAP. TRAP-positive cells appeared as dark red cells. TRAP-positive multinucleated cells containing more than three nuclei were counted as osteoclast-like cells. (TIF) [file pone.0085878.s001.tif]

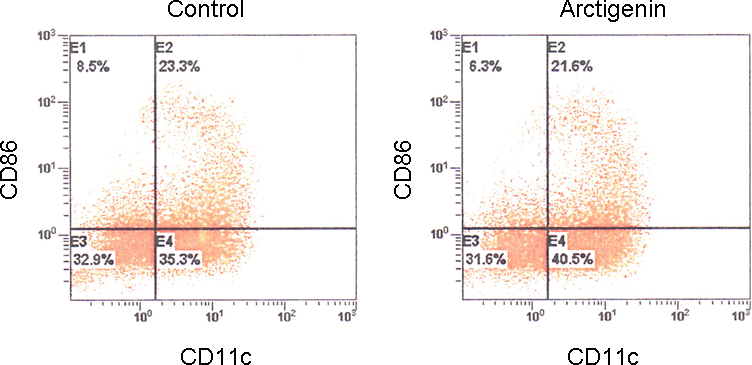

Supplement: Figure S2 — Effect of arctigenin on the differentiation of dendritic cells. BMMs (1.5×10^5^ cells) were cultured for 1 week in 60-mm dishes in the presence of 20 ng/mL GM-CSF with or without 1 µM arctigenin. Cells were analyzed for the expression of CD11c and CD86 by fluorescence-activated cell scanning. The numbers in the top right corners indicate the percentages of CD11c/CD86 double positive cells as differentiated dendritic cells. Experiments were performed four times, and representative data are shown. (TIF) [file pone.0085878.s002.tif]

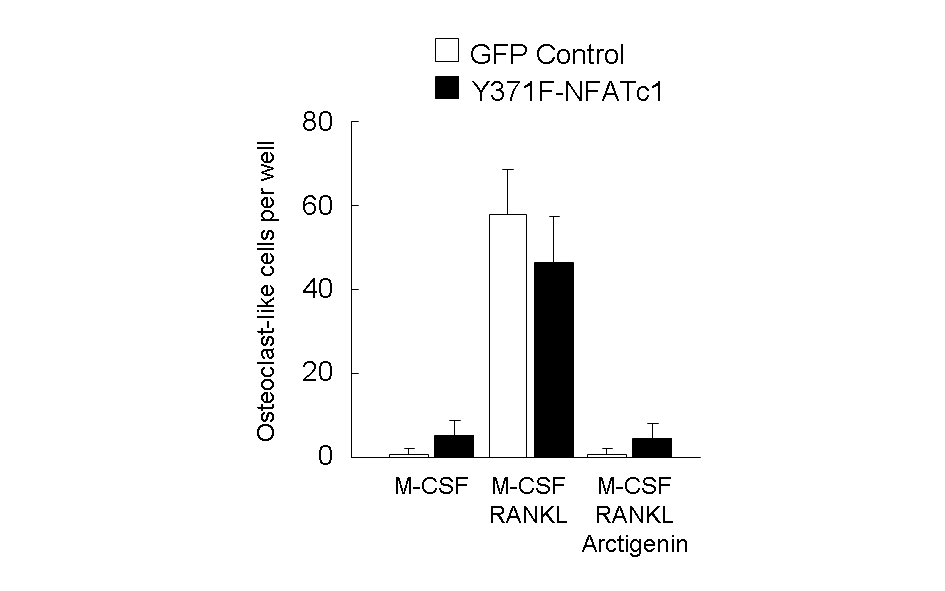

Supplement: Figure S3 — Effect of arctigenin on osteoclast-like cell formation induced by the forced expression of an Y371F-NFATc1 mutant. BMMs (3×10^4^ cells) were retrovirally transduced with Y371F-NFATc1 or control GFP, and cultured for 2 days in the presence of 5000 U/mL M-CSF in 96-well culture plates. Cells were further cultured in the presence of 100 ng/mL RANKL and/or 5000 U/mL M-CSF together with or without 1 µM arctigenin. After cultivation for 3 days, cells were fixed and stained for TRAP. TRAP-positive multinucleated cells containing more than three nuclei were counted as osteoclast-like cells. The results were expressed as means +/− SD (n = 4). (TIF) [file pone.0085878.s003.tif]

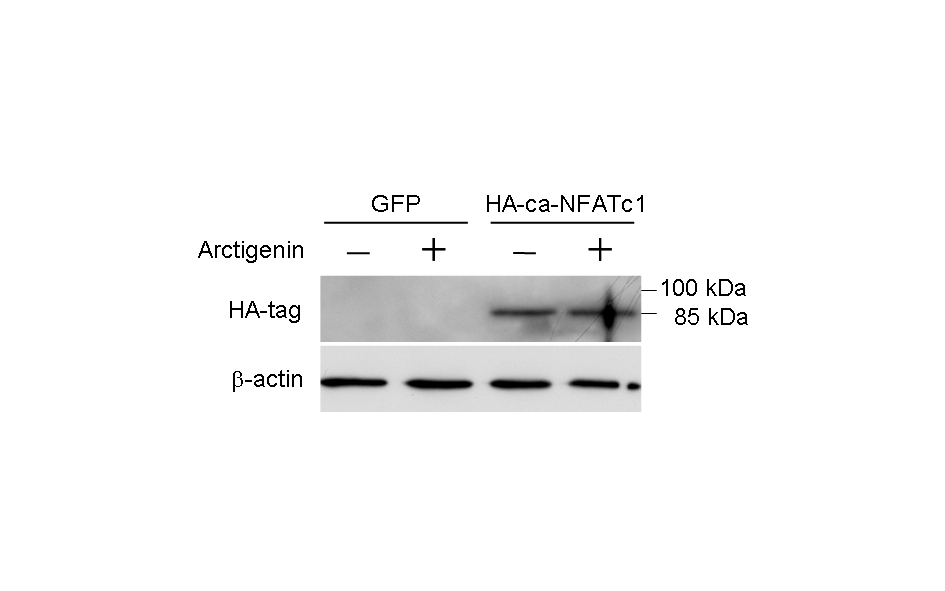

Supplement: Figure S4 — Effect of arctigenin on processing NFATc1. BMMs (3×10^4^ cells) were retrovirally transduced with a hemagglutinin (HA)-tagged ca-NFATc1 cDNA, and cultured for 2 days in the presence of 5000 U/mL M-CSF in 96-well culture plates. Cells were further cultured in the presence of 100 ng/mL RANKL and 5000 U/mL M-CSF together with or without 1 µM arctigenin. After cultivation for 2 days, total cell lysates were analyzed by Western blotting analysis using an anti-HA antibody or an anti-β-actin antibody. (TIF) [file pone.0085878.s004.tif]

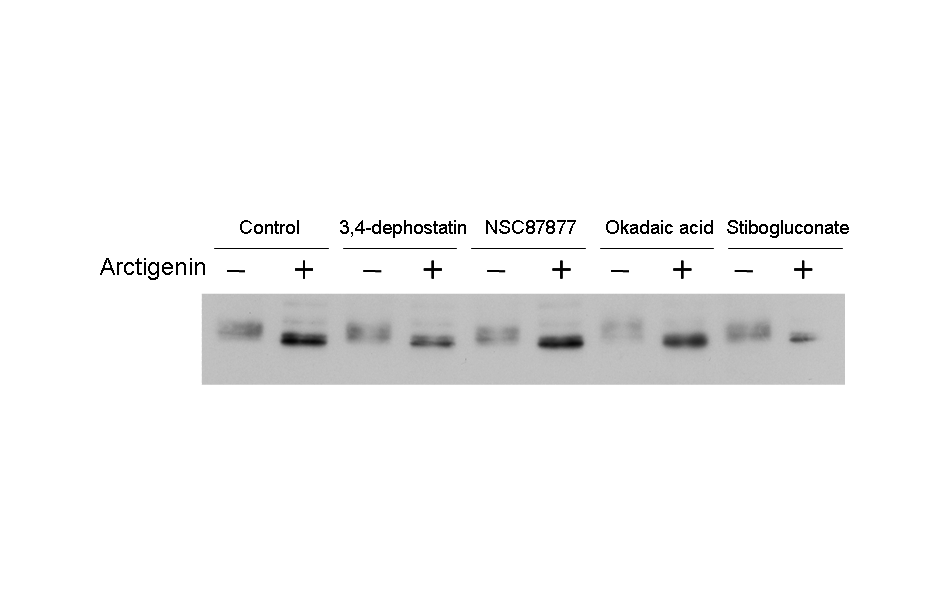

Supplement: Figure S5 — Effect of phosphatase inhibitors on the conversion of lower molecular species of NFATc1 induced by arctigenin. Purified osteoclast-like cells (2000 cells) were cultured in 24-well culture plates in the presence or absence of 10 µM 3,4-dephostatin [protein tyrosine phosphatase (PTP) inhibitor], 10 µM NSC87877 [SH2 domain-containing inositol phosphatase (SHIP)1/2 and PTP1B inhibitor], 0.5 µM okadaic acid (protein phosphatase 2A inhibitor), or 100 µM sodium stibogluconate (SHIP1 inhibitor) together with or without 1 µM arctigenin. After cultivation for 10 min, whole cell lysates were harvested and analyzed by Western blotting using an anti-NFATc1 antibody. (TIF) [file pone.0085878.s005.tif]

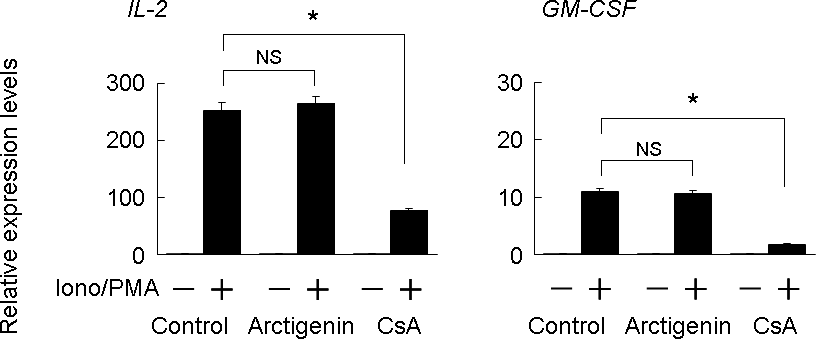

Supplement: Figure S6 — Effect of arctigenin on the expression of T cell-related genes. Mouse splenocytes (2×10^6^ cells) were activated for 4 h in the presence of 1 µM ionomycin and 20 nM PMA in 24-well culture plates. Cells were further cultured with or without 1 µM arctigenin and 1 µg/mL CsA. After cultivation for 2 h, IL-2 and GM-CSF mRNA levels were analyzed by quantitative RT-PCR. Expression levels were normalized to Gapdh and the values were relative to unstimulated controls. The results were expressed as means +/− SD (n = 3). *, p<0.05; NS, not significant. (TIF) [file pone.0085878.s006.tif]
